# College and Hospital Notes

**Published:** 1909-08

**Authors:** 


### COLLEGE AND HOSPITAL NOTES.

THE German Deaconess's Home, Buffalo, will soon have an addition to its present quarters on Kingsley street, to cost about $80,000, the plans for which having been drawn by Green and Wicks, the architects.

The new four-story addition to the Deaconess Home will extend back to Riley street, with an entrance on that street. It will give the home 75 new rooms and one of the finest operating-rooms of any hospital in the state. Around it will be built a balcony containing two rows of seats, from which students or visiting physicians may view operations without being in the way of the surgeons or nurses. The new building will be absolutely fire-proof. It is expected to break ground for the foundation this fall and the building operations will be in full swing by next summer.

JOHNS Hopkins University is considering plan to move to Homewood. It is considered that the $250,000 given to the Johns Hopkins University, May 27, 1909, by the Rockefeller general board of education in New York is the beginning of definite steps toward taking the University to Homewood, and is to be the nucleus of a fund of $1,000,000 to be raised as a condition of the gift, according to the statement made lately by R. Brent Keyser, president of the board of trustees of the institution. While the actual transfer of the university from its present site in the city to Homewood will not take place for many years, the announcement of this gift and the plans which are going ahead for taking several departments to Homewood show that the trustees are in earnest in fulfilling their oft-repeated desire to see the university on this site.

HARVARD University announced July 9, 1909, the establishment of a department in its Medical School exclusively devoted to public health and preventive medicine, and the election of Dr. Milton J. Rosenau, of Washington, as professor of hygiene and preventive medicine and head of the new department. Dr. Rosenau will begin his service at Harvard with the opening of the next college year.

THE Harrington Hospital for Children, made possible by the gift of a large part of the fortune of the late Dr. Devillo W. Harrington, was, dedicated May 27, 1909. The splendid new building, finished in every detail, was formally opened to the purposes for which Dr. Harrington designed it and for which it has been built. Several score invitations were sent out by the trustees and these were eagerly accepted. Owing to the limitations of the building the invitations could not be as general as the trustees desired.

